# An Alteration in the Lateral Geniculate Nucleus of Experimental Glaucoma Monkeys: *In vivo* Positron Emission Tomography Imaging of Glial Activation

**DOI:** 10.1371/journal.pone.0030526

**Published:** 2012-01-27

**Authors:** Masamitsu Shimazawa, Yasushi Ito, Yuta Inokuchi, Hajime Yamanaka, Tomohiro Nakanishi, Takuya Hayashi, Bin Ji, Makoto Higuchi, Tetsuya Suhara, Kazuyuki Imamura, Makoto Araie, Yasuyoshi Watanabe, Hirotaka Onoe, Hideaki Hara

**Affiliations:** 1 Molecular Pharmacology, Department of Biofunctional Evaluation, Gifu Pharmaceutical University, Gifu, Japan; 2 RIKEN Center for Molecular Imaging Science, Kobe, Japan; 3 Molecular Imaging Center, National Institute of Radiological Sciences, Chiba, Japan; 4 Department of Systems Life Engineering, Maebashi Institute of Technology, Maibashi, Japan; 5 Department of Ophthalmology, University of Tokyo School of Medicine, Tokyo, Japan; Universidade Federal do Rio de Janeiro, Brazil

## Abstract

We examined lateral geniculate nucleus (LGN) degeneration as an indicator for possible diagnosis of glaucoma in experimental glaucoma monkeys using positron emission tomography (PET). Chronic intraocular pressure (IOP) elevation was induced by laser trabeculoplasty in the left eyes of 5 cynomolgus monkeys. Glial cell activation was detected by PET imaging with [^11^C]PK11195, a PET ligand for peripheral-type benzodiazepine receptor (PBR), before and at 4 weeks after laser treatment (moderate glaucoma stage). At mild, moderate, and advanced experimental glaucoma stages (classified by histological changes based on the extent of axonal loss), brains were stained with cresyl violet, or antibodies against PBR, Iba-1 (a microglial marker), and GFAP (an activated astrocyte marker). In laser-treated eyes, IOP was persistently elevated throughout all observation periods. PET imaging showed increased [^11^C]PK11195 binding potential in the bilateral LGN at 4 weeks after laser treatment; the increase in the ipsilateral LGN was statistically significant (*P*<0.05, *n* = 4). Immunostaining showed bilateral activations of microglia and astrocytes in LGN layers receiving input from the laser-treated eye. PBR-positive cells were observed in LGN layers receiving input from laser-treated eye at all experimental glaucoma stages including the mild glaucoma stage and their localization coincided with Iba-1 positive microglia and GFAP-positive astrocytes. These data suggest that glial activation occurs in the LGN at a mild glaucoma stage, and that the LGN degeneration could be detected by a PET imaging with [^11^C]PK11195 during the moderate experimental glaucoma stage after unilateral ocular hypertension. Therefore, activated glial markers such as PBR in the LGN may be useful in noninvasive molecular imaging for diagnosis of glaucoma.

## Introduction

Open-angle glaucoma (OAG) is a slowly progressive and irreversible ocular disease that is one of the leading causes of blindness worldwide. Therefore, increasing attention is being paid to evaluation of the appearance of the optic nerve head and peripapillary retina in the diagnosis of OAG, especially in its early stage. Glaucoma pathology has been extensively studied at the level of the retinal ganglion cells (RGC) and optic nerve, with diagnosis resulting primarily from intraocular pressure (IOP), ophthalmoscopic, and visual field measurements. On the other hand, we and several investigators [1], [2], [3], [4] reported that neuronal degeneration in the lateral geniculate nucleus (LGN), which is the primary processing center for visual information received from the retina, occurred in experimental glaucoma models. Briefly, in experimental glaucoma monkeys, neuronal atrophy in the LGN appeared to occur at an early stage after IOP elevation, and LGN neurons were apparently more susceptible to the effects of IOP elevation than optic nerve axons [1], [2]. Furthermore, Gupta and her colleagues [5], [6] firstly reported that the death of the RGC in glaucoma patients is accompanied by transsynaptic degradation of neurons in the LGN. These findings strongly suggest that LGN degeneration may become new diagnostic and/or therapeutic targets for glaucoma.

Recently, we demonstrated that glial fibrillary acidic protein (GFAP) immunoreactivity as well as neuronal cell loss was increased in the LGN of cynomolgus monkeys at 4 months after unilateral ocular hypertension [7], and that microglial activation was detectable by positron emission tomography (PET) imaging with [^11^C]PK11195, a PET ligand for the peripheral-type benzodiazepine receptor (PBR), at 4 to 12 months after unilateral ocular hypertension [8]. PBR, also known as translocator protein (18 kDa) (TSPO) [9], is a highly hydrophobic protein located primarily on the outer mitochondrial membrane [10]. Although PBR is expressed at low or reduced levels in resting microglial cells and astrocytes of the normal brain, it is upregulated in activated microglial cells and astrocytes [11], [12], [13]. Thus, noninvasive molecular imaging of the LGN targeted against activated glial markers such as PBR may be helpful in the early diagnosis of glaucoma. However, the courses of glial activation and PBR expression that accompany LGN degeneration in glaucoma are as yet unknown.

In the present study, we examined LGN degeneration secondary to experimental hypertensive glaucoma as an indicator for glaucoma in ocular hypertensive monkeys using PET, and validated glial activation by morphological and immunohistochemical examinations.

## Materials and Methods

### Animals

The 5 adult cynomolgus monkeys (*Macaca fascicularis*) in this study, aged 4 to 6 years (Japan SLC Co. Ltd, Hamamatsu, Japan), were housed in an air-conditioned room at 24±2°C with 60±10% humidity, and given food and water *ad libitum*. The animal welfare and steps taken to ameliorate suffering were in accordance with the recommendations of the Weatherall report on the use of non-human primates in research, and all investigations were approved and monitored by the Institutional Animal Care and Use Committee of Kobe Institute in RIKEN (Permit Number: MAH18-05-15).

### Argon-Laser Photocoagulation (Experimental Glaucoma Model)

An elevation of intraocular pressure (IOP) was induced in each monkey by applying argon-laser burns to the mid portion of the trabecular meshwork of the left eye, with the right eye being used as an untreated control, as previously described [14], [15]. The laser irradiation was performed only on the left eyes, because we considered that laser irradiation on the ipsilateral eyes under the same conditions would facilitate accurate irradiation without technical dispersion. For the laser treatment, the animals were anesthetized with an intramuscular injection of ketamine (8.75 mg/kg, Ketalar 50®; Sankyo, Tokyo, Japan) plus xylazine (0.5 mg/kg, Celactal®; Bayer, Leverkusen, Germany). A single-mirror Goldmann lens (OSMGA, Ocular Instruments, Inc., Bellevue, WA, USA) filled with a hydroxyethylcellulose solution (Scopisol®15; Senjyu Pharmaceutical, Osaka, Japan) was then placed on the eye to be treated. An argon blue/green laser was focused on the mid-portion of the trabecular meshwork, and a total of 100 to 150 laser-beam spots was applied around 360° (spot size, 100 µm; power, 1000 mW; exposure time, 0.2 sec) using an argon-laser photo-coagulator (Ultima 2000 SE®; Coherent, Inc., CA, USA) attached to a standard slit-lamp microscope (BQ 900; Haag-Streit, Köniz, Switzerland). Two weeks after the first treatment, the laser treatment was repeated, except for monkey #5, so as to produce a maintained elevation in IOP. Time (in weeks) “after the laser treatment” was defined as dating from the first of these treatments.

### IOP and Ophthalmoscopy

IOP was measured in both eyes of each animal using a calibrated pneumatonometer (Model 30 Classic Pneumatonometer; Medtronic Solan, FL, USA) under ketamine anesthesia (8.75–10 mg/kg, i.m.), with local anesthesia using 0.4% oxibuprocaine hydrochloride (Benoxil® 0.4% solution; Santen Pharmaceutical Co. Ltd.). IOP was measured between 2 p.m. and 4 p.m. at 1- or 2-weeks intervals.

Then, fundus photographs (AFC-210; NIDEK Co. Ltd. Gamagori, Japan) were obtained under additive injection of xylazine (0.5 mg/kg, i.m.), and cup/disc (C/D) area ratio was assessed using data filing and analysis software (NAVIS-Lite ver 1.0.4.1, NIDEK Co. Ltd.). All measurements were taken at one- or two-week intervals.

### PET Imaging for [^11^C]PK11195

PET imaging was performed in 4 monkeys (#2 to #5) at 4 weeks after the first laser treatment, but monkey #1 was not measured the PET imaging, because monkey #1 showed IOP elevation, but did not change the optic disc. Before PET, a venous cannula was placed into a radial vain. For the scan, the animals were placed on the bed in a small animal PET scanner (microPET Focus220; Siemens Medical Solutions, Knoxville, TN, USA) under anesthesia with a continuous intravenous infusion of propofol (10 mg/kg/h, Hospira Japan Co. Ltd., Osaka, Japan). The spatial resolution at the center of the field of view was ∼1.35 mm in full width at half maximum (FWHM). The [^11^C]PK11195 was prepared according to previously described methods, using the reaction of (R)-*N*-desmethyl precursor (1 mg) in dimethylsulfoxide (200 µl) containing 1 mg of KOH, with [^11^C]-CH_3_I produced by a cyclotron (HM12; Sumitomo Heavy Industry, Tokyo, Japan) [16]. Radiochemical purities were >99.5%, and the mean (± SD) specific activity was 65.9 (±29.4) GBq/µmol. After a transmission scan for 30 min with ^68^Ga-^68^Ge pin source, [^11^C]PK11195 (187.6±22.9 MBq) was injected as an intravenous bolus, and PET scanning was performed for 90 min. PET images were acquired with three-dimensional list mode reconstructed by an algorithm of filtered back projection (FBP), and were smoothed by a Hanning filter with a cutoff of 0.4. Attenuation correction, but not scatter fraction correction, using blank and transmission images, was performed to obtain quantitative images. A dynamic histogram acquired during 90 min (6×10 sec, 6×30 sec, 11×60 sec, 15×180 sec, 3×600 sec) was used for data analysis.

Reconstructed images were processed with PMOD version 3.0 image analysis software (PMOD Technologies, Zürich, Switzerland). To elucidate the quantitative [^11^C]PK11195 binding to PBR in the brain, the cerebellum was used as a reference region to calculate binding potential value in a simplified reference tissue model (SRTM) developed by Lammertsma and Hume [17]. Regional binding potential values were obtained by applying some regions of interest (ROI) to the individual PET images aligned on individual magnetic resonance imaging (MRI). ROIs were manually drawn with reference to the stereotaxic brain atlas of the cynomolgus monkey for the cerebellum and LGN.

MRI was performed for all animals using a 3-T MRI scanner (Signa Horizon Lx VH3; General Electric Healthcare, Milwaukee, WI) under pentobarbital anesthesia to define anatomical ROIs in a separate session from PET imaging.

### Histological Examination

After the last measurements, animals were transcardially perfused to the bilateral common carotid arteries with 2.0 liters of 0.9% saline containing 10 U/ml heparin at 4°C after ligation of thoracic aorta., under deep sodium pentobarbital anesthesia (30 mg/kg, i.v.; Nembutal; Abbott, North Chicago, IL, USA), followed by 2.0 liters of 4% paraformaldehyde in 0.1 M phosphate buffer (pH 7.4). The brains and eyeballs with optic nerve were removed after the perfusion. The brains and eyes were cut into several sections and immersed in the same fixative solution for at least 24 h, soaked in 10 to 30% (w/v) sucrose, then frozen in embedding compound (Tissue-Tek; Sakura Finetechnical Co. Ltd., Tokyo, Japan). Next, 20-µm thick coronal sections of the LGN and retina were serially cut, then stained with cresyl violet or hematoxylin and eosin, respectively. Retinal damage was evaluated as follows: six sections stained with hematoxylin and eosin from each eye being used for the morphometric analysis. Light-microscope photographs were taken using a digital camera a charge-coupled device camera (MicroPublisher 5.0RTV, QIMAGING, Burnaby, BC, Canada), and the cell counts in the ganglion cell layer (GCL) and inner nuclear layer (INL) at a distance between 1750 and 2200 µm from the optic disc were measured on the images in a masked fashion by a single observer (Y. I.). Data from six sections were averaged for each eye, and the values obtained were used to evaluate the GCL and INL cell counts.

To assess number and size of neurons in the LGN, sections stained with cresyl violet were used for these measurements: The measurements were performed on three sections representative of the anterior, middle, and posterior parts of each LGN. The volume used for the measurements was 216×162×20 µm (section thickness) of each side in the LGN. Care was taken to count only neurons with clearly visible nuclei and cytoplasm. For quantification of cresyl violet-stained sections, neuron numbers in the M- and P-layers were estimated by counting under objective ×40 with brightfield microscope in a masked fashion by a single observer (Y.I.). The number on each section was measured using image-processing software (Image-J ver. 1.33f; National Institutes of Health, USA). Neuron counts were derived from three regions per layer in each of the three LGN sample sections. The effects of elevated IOP on neuronal cell size were studied by measuring 1200 geniculate neurons in each animal. Neuron samples (100 neurons/layer) were taken randomly from the full width of each lamina and included only those neurons with clearly visible nucleoli, and measured soma area. Average neuronal cell size for each layer was calculated on the basis of data from 100 neurons.

Segments of optic nerve from 3 mm behind the eye were postfixed by immersion in 1.25% glutaraldehyde with 2% paraformaldehyde in PBS for at least 1 week at 4°C. After three washes with PBS, the nerve segments were placed in 2% osmium tetroxide in saline for 2 h, then washed with PBS at room temperature. Subsequently, they were dehydrated in alcohol and embedded as cross-sections in epoxy resin for sectioning. Cross-sections (1 µm thickness) were cut with an ultramicrotome, mounted on glass slides, and stained for myelin using 1% toluidine blue. For counting of the numbers of RGC axons, the optic nerve area was divided into five segments (nasal, temporal, superior, inferior and central) in each optic nerve sections. We captured thirteen images from these segments in each optic nerve at high power using an oil immersion objective ×100 with bright-field microscope completed with wide field and high resolution CCD camera (BZ-9000, KEYENCE CORPORATION, Osaka, Japan),. In each image (145×109 µm), every myelinated axon was counted in masked fashion by a single observer (T.N.) and the data from the thirteen images were summed. Estimated total area was covered more than 3% per nerve, according to the previous report [18]. Axon number was expressed as (healthy axon number on left side)/(healthy axon number on right side)×100% for each animal. Glaucoma stages were classified by the extent of axonal loss as mild (0–20%), moderate (20–50%), and advanced (50–100%) (Table 1).

**Table 1:** Changes in IOP and the optic disc in cynomolgus monkeys after laser-photocoagulation.

				Glaucomatous left eye					
No.	Baseline		D	Mean	Max.	Integral	C/D area		Glaucoma
	IOP		(weeks)	IOP	IOP	ΔIOP	ratio		stages
	(mmHg)			(mmHg)	(mmHg)		(left eye)		
	R	L					Pre	End	
#1	22.6	24.3	9	40.2	52.7	1109.5	0.19	0.20	Mild
#2	21.7	23.4	4	61.3	72.0	475.7	0.25	0.40	Moderate
#3	21.0	20.3	11	60.2	73.3	2501.9	0.17	0.64	Advanced
#4	24.3	24.7	15	62.1	70.7	3267.8	0.19	0.56	Advanced
#5	24.8	22.4	24	50.8	60.0	4450.8	0.23	0.77	Advanced

C/D area ratio: cup/disc area ratio, ONH: Optic nerve head, IOP: Intraocular pressure, R: right eye, L: left eye, D: Duration, Max IOP: Maximum IOP, Integral ΔIOP: Integral IOP to right eye (Δ mmHg/day). Glaucoma stages were classified by histological changes based on the extent of axonal loss as mild (0–20%), moderate (20–50%), and advanced glaucoma (50–100%).

### Immunohistochemistry

During the immunostaining procedures, coronal sections containing LGN were washed with 0.01 M phosphate-buffered saline (PBS, pH 7.4), then treated with 0.3% hydrogen peroxide in 0.01 M PBS. They were then preincubated with 10% normal goat serum (Vector, Burlingame, CA, USA) in 0.01 M PBS for 30 min and incubated for one day at 4°C with specific mouse anti-glial fibrillary acidic protein (GFAP) monoclonal antibody (1∶800 dilution) (MAB360; Chemicon, Temecula, CA, USA), rabbit anti-ionized calcium-binding adaptor molecule 1 (Iba-1) polyclonal antibody (1: 600 dilution) (019-19741;Wako, Osaka, Japan) in a solution of 10% normal goat serum in 0.01 M PBS containing 0.3% (v/v) Triton X-100. The coronal sections were then washed with PBS and incubated with biotinylated anti-mouse or anti-rabbit IgG before being incubated with avidin-biotin-peroxidase complex for 30 min at room temperature. Finally, diaminobenzidine was used as a peroxidase substrate for visualization. To assess area of GFAP and Iba-1 immunoreactivity in the LGN, sections immunostained with GFAP or Iba-1 were used for the area measurements: The measurements were performed on three sections representative of the anterior, middle, and posterior parts of each LGN. The volume used for the area measurements was 216×162×20 µm (section thickness) of each side in the LGN. The measurements were carried out under objective ×40 with brightfield microscope in a masked fashion by a single observer (Y.I.). The area on each section was measured using MetaMorph software (Molecular Devices, Sunnyvale, CA , USA). Data from each section was averaged for each LGN, and the values obtained were used to evaluate the area of GFAP and Iba-1 immunoreactivity.

### Immunofluorescence Staining

During the immunofluorescence staining procedures, coronal sections containing LGN were subjected to antigen retrieval by autoclaving in 0.01 M sodium citrate buffer, pH 6.0, at 121°C for 15 min. Next, they were preincubated with 10% normal goat serum (Vector) in 0.01 M PBS for 30 min, then incubated for one day at 4°C with specific rabbit anti-peripheral benzodiazepine receptor (PBR) polyclonal antibody (1∶1000 dilution) in a solution of 10% normal goat serum in 0.01 M PBS containing 0.3% (v/v) Triton X-100. We raised a rabbit polyclonal antibody against the peptide residues 36–47 of mouse PBR (SLQKPSWHPPRW), designated NP36, and purified it with an antigen peptide affinity-column to ensure reactivity against the antigen. After washing with 0.01 M PBS, the sections were incubated for 3 h at room temperature with a mixture of an Alexa Fluor 488 F(ab′)_2_ fragment of goat anti-rabbit IgG (H+L) (1∶1000 dilution) (A11070; Invitrogen, Carlsbad, CA, USA). To assess number of PBR-positive cell in the LGN, sections immunostained with PBR were used for the number measurement: The Measurement were performed on three sections representative of the anterior, middle, and posterior parts of each LGN. The volume used for the measurements was 216×162×20 µm (section thickness) of each side in the LGN. The measurements were carried out under objective ×40 with brightfield microscope in a masked fashion by a single observer (Y.I.). The number on each section was measured using image-processing software (Image-J ver. 1.33f; National Institutes of Health, USA). Data from each section was averaged for each LGN, and the values obtained were used to evaluate the number of PBR-positive cell.

Retinal sections were preincubated with 10% normal horse serum and (Vector) and 0.3% TritonX100 in 0.01 M PBS for 30 min, then incubated for one day at 4°C with mouse anti-calbindin mAb (1∶200 dilution) (Abcam, Cambridge, MA, USA), rabbit anti-calretinin mAb (1∶200 dilution) (Abcam), mouse anti-parvalbumin mAb (1∶1000 dilution) (Millipore, Temecula, CA, USA) or and goat anti-Brn3a (1∶1000 dilution) (Santa Cruz Biotechnology, Santa Cruz, CA, USA), in a solution of 10% normal horse serum in 0.01 M PBS containing 0.3% (v/v) Triton X-100. After washing with 0.01 M PBS, the sections were incubated for 3 h at room temperature with a mixture of AlexaFluor 546 labeled rabbit anti-goat IgG (1∶1000 dilution) (Invitrogen) and AlexaFluor 488 labeled rabbit anti-mouse IgG(F(ab′)_2_) (1∶1000 dilution) (Invitrogen), or with a mixture of AlexaFluor 546 labeled donkey anti-rabbit IgG (1∶1000 dilation) (Invitrogen) and AlexaFluor 488 labeled donkey anti-goat IgG (1∶1000 dilution) (Invitrogen) in PBS with 10% normal horse serum and 0.3% TritonX100 and Hoechst33342 (1∶5000 dilution) (Invitrogen) for 3 h at room temperature. Immunofluorescence images were taken using a microscope (BX50; Olympus, Tokyo, Japan) with a cooled charge-coupled device camera (DP30BP; Olympus) at 1360×1024 pixels via MetaMorph software (Molecular Devices), and the calbindin-positive cells were counted in the inner part of inner nuclear layer (INL) at a distance between 1750 and 2200 µm from the optic disc on the images in a masked fashion by a single observer (M.S.).

### Double Immunofluorescence Staining

To confirm the cell phenotypes containing PBR, we performed double immunofluorescence staining for PBR and GFAP or Iba-1 on LGN sections. Coronal sections containing LGN were prepared as described above.

To visualize co-localization of PBR with GFAP, the sections were incubated overnight at 4°C with rabbit anti-PBR polyclonal antibody (1∶1000 dilution) and mouse anti-GFAP monoclonal antibody (1∶800 dilution) in a solution of 10% normal goat serum in 0.01 M PBS with 0.3% (v/v) Triton X-100. Next, they were washed with 0.01 M PBS and then incubated for 3 h at room temperature with a mixture of an Alexa Fluor 488 F(ab′)_2_ fragment of goat anti-rabbit IgG (H+L) (1∶1000 dilution) (Molecular Probes) and an Alexa Fluor 546 F(ab′)_2_ fragment of goat anti-mouse IgG (H+L) (1∶1000 dilution) (A-11018; Molecular Probes). Co-localization of PBR with Iba-1 was visualized by double immunofluorescence staining on LGN sections. The sections were incubated with a primary antibody for PBR (1∶500 dilution) labeled with Zenon Alexa Fluor 488 (Z25302; Molecular Probes), and with a primary antibody for Iba-1 (1∶300 dilution) with Zenon Alexa Fluor 594 (Z25207; Molecular Probes,) for 3 h at room temperature.

### Data Analysis

Data are expressed as mean ± S.E.M. A paired *t*-test was used to compare [^11^C]PK11196 binding potential between before and 4 weeks after the laser treatment. Pearson correlation coefficient was calculated between the mean PBR-positive cell numbers in LGN layers receiving input from glaucoma eye and the percent changes of GFAP-, Iba-1-immunoreactivities, soma size in LGN, or percent change of axon numbers in optic nerve in each of five glaucoma monkeys. Results were considered significantly different if *P*<0.05.

## Results

### Fundus Images and Retinal Damage after IOP Elevation

**Figure 1:**
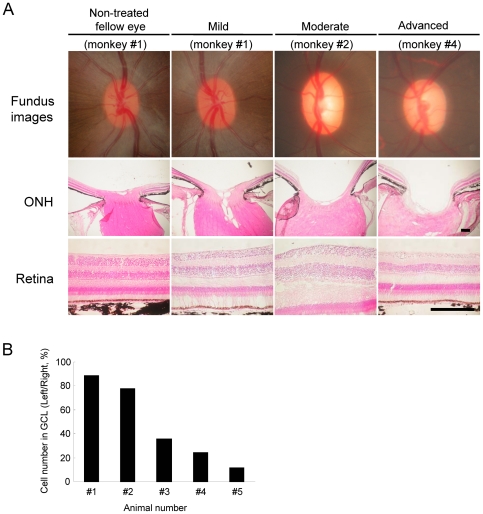
Ocular fundus photographs and histological sections of the retina at each experimental glaucoma stage in laser-treated eyes of cynomolgus monkeys. (A) Ocular fundus photographs were taken just before the end of the experiment for each monkey Each scale bar indicates 200 µm for ONH and retina. (B) The cell numbers in ganglion cell layers (GCL) of each experimental glaucoma stage were estimated by counting GCL cells at a distance between 1750 and 2200 µm from the optic disc. Cell number was expressed as (healthy cell number on GCL of left eye)/(healthy cell number on GCL of right eye)×100% for each animal.

**Figure 2:**
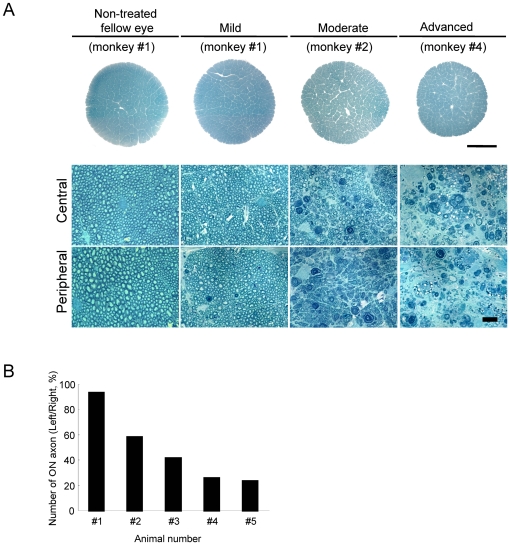
Optic nerve degeneration at each experimental glaucoma stage in laser-treated eyes of cynomolgus monkeys. (A) Optic nerve were cut with an ultramicrotome, mounted on glass slides, and stained for myelin using 1% toluidine blue. Each scale bar indicates 1000 µm (low magnification images) and 10 µm (high magnification images), respectively. (B) The axonal numbers in optic nerve (ON) of each experimental glaucoma stage were estimated by counting myelinated axons in each segment 145×109×1 µm (section thickness). Axon number was expressed as (healthy axon number on left side)/(healthy axon number on right side)×100% for each animal.

The mean IOP value in 10 eyes of 5 normal cynomolgus monkeys examined in this study was 22.9±0.7 mmHg (*n* = 5) in the right eye and 23.0±0.8 mmHg (*n* = 5) in the left eye. The IOP data are summarized in Table 1. There were no significant changes (*p*>0.05) between the two eyes. The IOP of the monkeys was elevated and remained above baseline throughout the observation period (4–24 weeks) after the first laser treatment. The extent of IOP elevation was similar among monkey #2 to #5, but it was lower in monkey #1 than other 4 monkeys. No obvious retinal lesions, nerve fiber layer defects, or abnormalities of either the optic nerve head or vascular structures were observed in any of the eyes before laser irradiation. Ocular fundus photographs of laser-treated eyes at each glaucoma stage are shown in Fig. 1A. Optic disc cupping was seen and increased depending on the extent of IOP elevation and duration after laser irradiation (Fig. 1A and Table 1). The cell numbers in GCL and myelinated axon in the optic nerve (ON), but not the cell number in INL, were also decreased depending on the extent of IOP elevation and duration (Figs. 1B, 2 and Table 2): the cell number of GCL was 88.5, 77.9, 35.8, 24.0, and 11.7% of that in the contralateral control GCL of monkey #1 to #5, respectively, the cell number of INL was 102.3, 104.8, 97.9, 99.1, 98.9% of that in the contralateral control INL of monkey #1 to #5, respectively , and the mean axonal number in the (left) ON was 93.7, 58.9, 42.2, 26.8, and 24.4% of that in the contralateral control (right) ON of monkey #1 to #5, respectively. On the other hand, a monkey #1 defined as mild glaucoma stage (Table 1) showed a persistent IOP elevation, but there was slight optic disc change based on ophthalmoscopy during 9 weeks. Therefore, histological analysis was performed in this monkey at 9 weeks after the laser treatment, and focal degeneration of the optic nerve was observed at the peripheral region (89.1% of the axon number in the fellow ON), but not at the central region (102.4% of the axon number in the fellow ON) (Fig. 2).

**Table 2:** Pathological changes in cynomolgus monkeys after laser-photocoagulation.

No.	Glaucoma	RGC	Neuronal loss	Immunoreactivity in LGN layers		
	stages	loss	in LGN layers	GFAP	Iba-1	PBR
#1	Mild	+	−	+	+	+++
#2	Moderate	++	+	+++	++	+++
#3	Advanced	+++	+++	+++	+++	+++
#4	Advanced	+++	+++	+++	+++	+++
#5	Advanced	+++	+++	+++	++	+++

LGN: lateral geniculate layer, ONH: optic nerve head, ON: optic nerve, GFAP: glial fibrillary acidic protein, Iba-1: ionized calcium-binding adaptermolecule-1, PBR: peripheral benzodiazepine receptor. Glaucoma stages were classified by histological changes based on the extent of axonal loss as mild (0–20%), moderate (20–50%), and advanced glaucoma (50–100%). Neuronal atrophy, RGC loss and immunoreactivity were classified based on the quantitative data as follows: no changes; −, mild; +, moderate; ++, robust; +++.

### [^11^C]PK11195 Binding in LGN before and during the moderate stage of glaucoma after Laser Treatment

**Figure 3:**
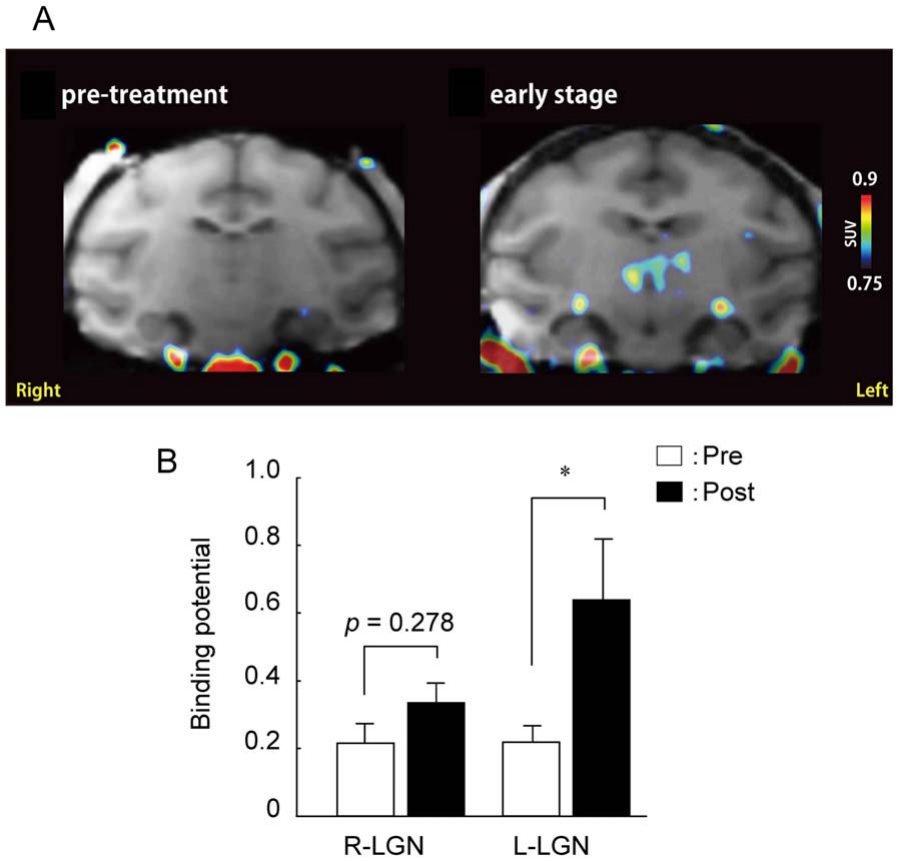
Changes in optic disc and [^11^C]PK11195 PET image in coronal slices from cynomolgus monkeys at a moderate stage after laser photocoagulation. (A) Accumulation of [^11^C]PK11195 binding potential in the LGN before and 4 weeks after laser photocoagulation. Coronal planes at the level of the LGN are shown. The PET images are shown in color, overlaid on the template MRIs is shown in gray scale. The color scale on the right represents standard uptake value (SUV). (B) Binding potential values of the right and left LGN obtained from 4 different individuals (4 monkeys; #2, #3, #4, #5, seen in tables 1 and 2). White and black column indicate pretreatment (pre) and post treatment (post) periods, respectively, for the laser photocoagulation. Each column represents the mean ± S.E.M. (*n* = 4). *p<0.05 versus the pretreatment.

We measured binding potential value of [^11^C]PK11195 in the brain before and during the moderate stage of glaucoma based on ophthalmoscopy (at 4 weeks) after the laser treatment in 4 cynomolgus monkeys. Representative PET images in a moderate glaucoma monkey are shown in Fig. 3A. At the moderate stage of glaucoma, mean cup/disc area ratio was 0.43±0.02 (monkeys #2 to #5, *n* = 4) against 0.21±0.02 (*n* = 4) before the laser treatment. In the PET study, [^11^C]PK11195 binding potential value was significantly increased in the ipsilateral LGN during the moderate glaucoma stage after unilateral ocular hypertension compared to before the laser treatment (Fig. 3B). In contrast, in the contralateral LGN, [^11^C]PK11195 binding potential value tended to increase but the differences were not statistically significant (*p* = 0.278, Fig. 3B).

### Expression of the Peripheral Benzodiazepine Receptor (PBR) in the LGN at Each Experimental Glaucoma Stage

**Figure 4:**
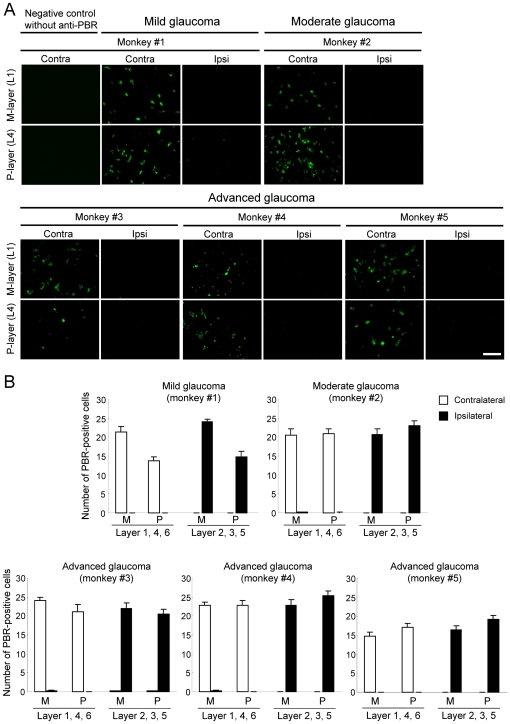
The expression of peripheral benzodiazepine receptor (PBR) in LGN layers receiving input from a glaucomatous eye. Each experimental glaucoma stage was classified by the glaucoma severity based on the extent of axonal loss as mild (0–20%), moderate (20–50%), and advanced (50–100%). (A) Representative photographs of PBR-positive cells in LGN layers of the contralateral (Contra) and ipsilateral (Ipsi) sides are shown for each experimental glaucoma stage. Layers 1 (L1) and 4 (L4) on the contralateral side receive their innervations from the glaucomatous (left) eye, whereas L1 and L4 on the ipsilateral side receive their retinal inputs from the non-treated fellow (right) eye. Each scale bar indicates 50 µm. (B) The numbers of PBR-positive cells on each section were measured, and averaged for each LGN as mean ± S.E.M. at each M-(layers 1 and 2) and the P-(layers 3–6) layers.

To examine the expression of PBR in the LGN at each experimental glaucoma stage, we used antibodies against PBR (Fig. 4). PBR-positive cells were observed in the LGN layers (including both magnocellular- and parvocellular-layers) receiving input from laser-treated eyes at all experimental glaucoma stages, and PBR was expressed even at the mild glaucoma stage (Fig. 4 and Table 2). On the other hand, no PBR-positive cells were observed in the LGN layers receiving input from non-treated fellow eyes. Furtheremore, there was no fluorescence detected from negative controls without anti-PBR antibody (Fig. 4A).

**Figure 5:**
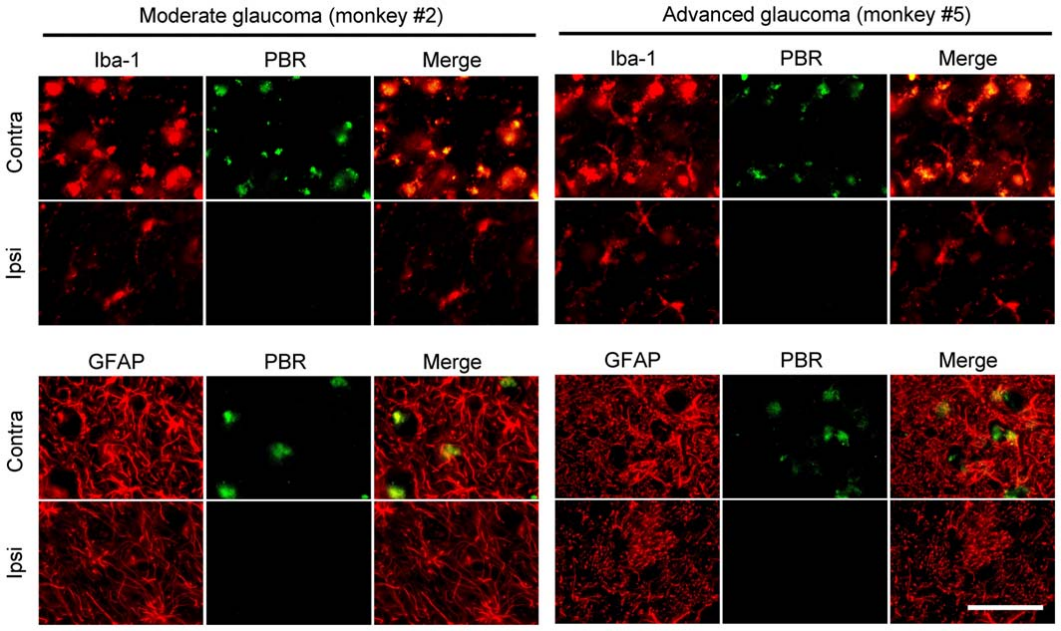
Double-immunofluorescence staining for PBR with a microglial marker (Iba-1) or a reactive astrocyte marker (GFAP). Layer 1 (L1) on the contralateral side receive their innervations from the glaucomatous (left) eye, whereas L1 on the ipsilateral side receive their retinal inputs from the non-treated fellow (right) eye. Each scale bar indicates 50 µm.

To identify PBR-positive cells in the LGN, double immunofluorescence staining was performed for PBR and Iba-1 or for PBR and GFAP (Fig. 5). PBR-positive cells were co-labeled in a portion of the Iba-1-positive microglial cells and GFAP-positive astroglial cells in the LGN at both moderate (monkey #2) and advanced glaucoma (monkey #5) stages (Fig. 5).

### Histological Changes and Glial Activation in LGN at Each Experimental Glaucoma Stage

**Figure 6:**
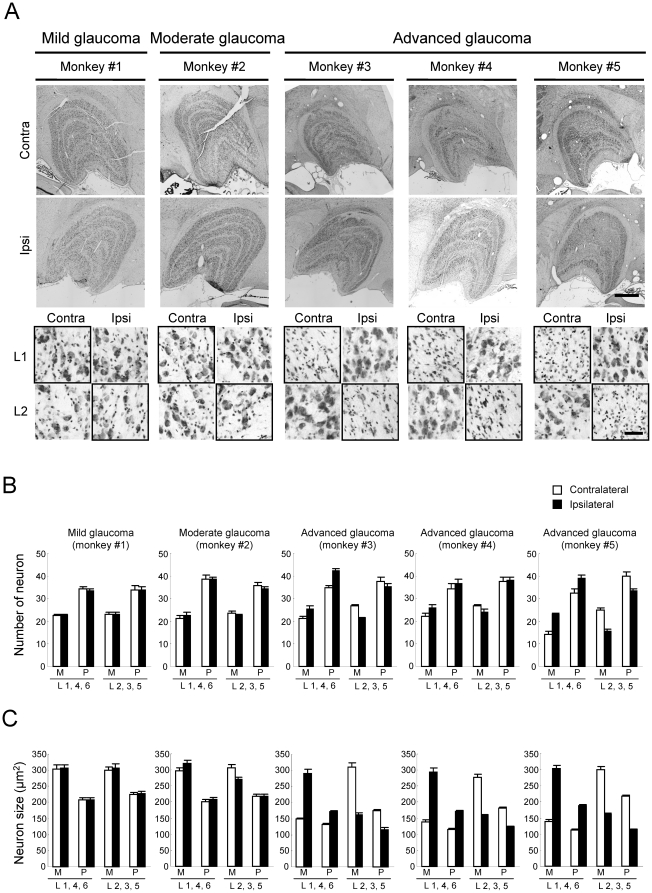
Histological changes in LGN neurons of cynomolgus monkeys after laser photocoagulation. (A) Representative microphotographs of cresyl violet-stained coronal sections of LGN on the contralateral and ipsilateral sides for each experimental glaucoma stage. Each experimental glaucoma stage was classified by the glaucoma severity based on the extent of axonal loss as mild (0–20%), moderate (20–50%), and advanced (50–100%). Layer 1 on the contralateral side and layer 2 on the ipsilateral side receive their innervations from the glaucomatous (left) eye (shown in bold frame), whereas layer 1 on the ipsilateral side and layer 2 on the contralateral side receive their retinal inputs from the non-treated fellow (right) eye. L1 and L2: layer 1 and 2. Scale bars indicate 1000 µm in whole LGN image and 50 µm in enlarged image. Neuron number (B) and mean neuronal cell size (C) in each layer of LGN were measured and averaged as mean ± S.E.M. at each M-(layers 1 and 2) and the P-(layers 3–6) layers.

**Figure 7:**
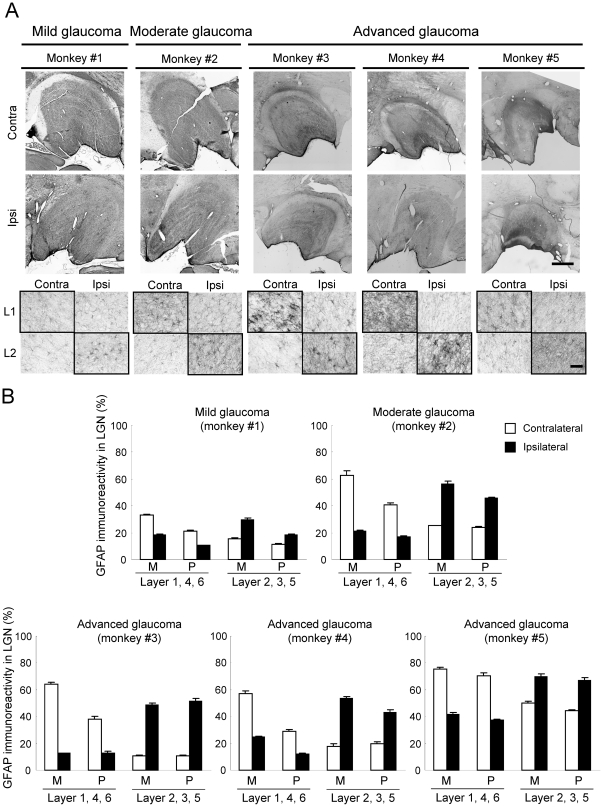
Astrocyte activation in LGN layers receiving input from a glaucomatous eye. (A) Representative photographs of GFAP immunoreactivity in LGN of the contralateral and ipsilateral sides are shown for each experimental glaucoma stage. Each experimental glaucoma stage was classified by the glaucoma severity based on the extent of axonal loss as mild (0–20%), moderate (20–50%), and advanced (50–100%). Layer 1 on the contralateral side and layer 2 on the ipsilateral side receive their innervations from the glaucomatous (left) eye (shown in bold frame), whereas layer 1 on the ipsilateral side and layer 2 on the contralateral side receive their retinal inputs from the non-treated fellow (right) eye. L1 and L2: layer 1 and 2. Scale bars indicate 1000 µm in whole LGN image and 50 µm in enlarged image. (B) The GFAP-immunoreactive area on each section was measured, and averaged for each LGN as mean ± S.E.M. at each M-(layers 1 and 2) and the P-(layers 3–6) layers.

**Figure 8:**
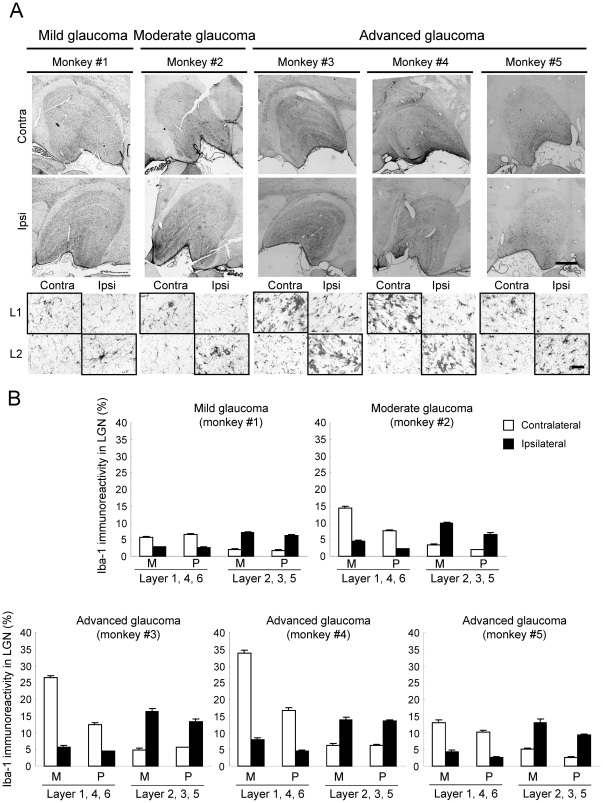
Microglial activation in LGN layers receiving input from a glaucomatous eye. (A) Representative photographs of Iba-1 immunoreactivity in LGN of the contralateral and ipsilateral sides are shown for each experimental glaucoma stage. Each experimental glaucoma stage was classified by the glaucoma severity based on the extent of axonal loss as mild (0–20%), moderate (20–50%), and advanced (50–100%). Layer 1 on the contralateral side and layer 2 on the ipsilateral side receive their innervations from the glaucomatous (left) eye (shown in bold frame), whereas layer 1 on the ipsilateral side and layer 2 on the contralateral side receive their retinal inputs from the non-treated fellow (right) eye. L1 and L2: layer 1 and 2. Scale bars indicate 1000 µm in whole LGN image and 50 µm in enlarged image. (B) The Iba-1-immunoreactive area on each section was measured, and averaged for each LGN as mean ± S.E.M. at each M-(layers 1 and 2) and the P-(layers 3–6) layers.

To examine the histological and pathological changes in the LGN at each stage of experimental glaucoma, coronal sections including the LGN were stained with cresyl violet, and for antibodies against PBR, ionized calcium-binding adaptor molecule 1 (Iba-1; a microglial marker), and glial fibrillary acidic protein (GFAP; an activated astrocyte marker) (Figs. 6, 7, 8 and Table 2). Cresyl violet staining showed that the number of neuron and neuronal size in the LGN layers (1, 4 and 6 on the contralateral, and 2, 3 and 5 on the ipsilateral) receiving input from the laser-treated eyes were decreased at advanced glaucoma stages (monkeys #3, #4, and #5), but no changes were seen in the LGN layers receiving input from the fellow eye (Fig. 6). No morphological changes including cell number and cellsize were seen in any LGN layers receiving input from eye in the mild glaucoma (monkey #1), but neuronal size was slightly decrease in the magnocellular (M) layer, but not parvocellular (P-) layer, of LGN receiving input from eye in the moderate glaucoma (monkey #2) stage (Fig. 6).

We next tested glial activation in the LGN after laser irradiation (Figs. 7, 8 and Table 2). Numbers of GFAP-positive astroglial cells (Fig. 7) and Iba-1-positive microglial cells (Fig. 8) were increased in the LGN layers receiving input from laser-treated eyes compared to the LGN layers receiving input from the fellow eyes. These changes depended on the extents of RGC and optic nerve losses after laser irradiation (Figs. 1, 2 and Table 2). Even at the mild glaucoma stage, GFAP-positive astroglial cells and Iba-1-positive microglial cells were increased in the bilateral LGN (Figs. 7 and 8).

Next, we performed Pearson correlation coefficient analysis between PBR and GFAP, Iba-1, LGN degeneration (soma size), or optic nerve axons (a glaucoma severity) in each of five glaucoma monkeys. The correlation coefficients between the mean PBR-positive cell numbers in LGN layers receiving input from glaucoma eye and percent changes of GFAP-, Iba-1-immunoreactivity, or soma size in LGN, or optic nerve axons were 0.512 (*P* = 0.378, *n* = 5), 0.064 (*P* = 0.918, *n* = 5), −0.199 (*P* = 0.748, *n* = 5), and −0.426 (*P* = 0.475, *n* = 5), respectively. In these comparisons, some parameters showed higher correlation coefficients of 0.512 (between PBR and GFAP) and −0.426 (between PBR and optic nerve axons) than other parameters, but did not show any significance. On the other hand, there was a significant correlation (r = 0.923, *P* = 0.025, *n* = 5) between percent changes of axon numbers in optic nerve and soma size in LGN.

## Discussion

In the present study, we demonstrated that binding potential value for [^11^C]PK11195 was increased in the LGN of unilateral hypertensive monkeys at a moderate experimental glaucoma stage (at 4 weeks after laser treatment). PBR was persistently expressed in glial cells of the LGN receiving input from laser-treated eye in mild through advanced stages of experimental glaucoma.

In experimental glaucoma, axonal injury in the lamina results in rapid Wallerian degeneration of the distal axon prior to the death of the RGC cells [19]. Howell et al. [20] have also reported that the distal axons are more vulnerable than the RGC soma and proximal axons in a mouse model of glaucoma (DBA/2J). In the present study, a mild glaucoma monkey with slight increase of disc cupping and a moderate glaucoma monkey with mild increase of disc cupping showed mild and moderate axonal degeneration, respectively, in the optic nerve, but RGC losses were a modest. Thus, the axonal injury is more severe than RGC loss during moderate glaucoma stage. These data strongly support the idea that the axonal injury is an initial key step in glaucoma, and therefore the loss of the RGC soma may be preceded by axonal degeneration. The RGC is known to die by apoptosis in glaucoma [21], [22], but the precise mechanism underlying the transsynaptic degeneration of the retinogeniculate pathways remains unknown.

Recently, evidences have been growing that the atrophy of the LGN neuron occurs in experimental primate [1], [2], [3] and human glaucoma [5], [6]. In primate glaucoma, we and others have reported that the neuronal shrinkage within LGN occurs at an early phase after IOP elevation [1], [2], [3]. In the present study, there was a significant correlation (r = 0.923, *P* = 0.025) between axonal degeneration in optic nerve and neuronal atrophy in LGN. These findings indicate that the decreased input from the retina to LGN is caused by optic nerve dysfunction and/or degeneration. This type of neurodegeneration starts at the axon terminals and progresses to the cell bodies. In the present study, activation of microglia and astrocytes was observed bilaterally in the LGN layers receiving input from the treated eye and even at the mild glaucoma stage. These results were also consistent with our previous reports that showed immunoreactivity with GFAP and major histocompatibility complex Class II CR3/43 [23], which are antigens specifically expressed in activated microglia, were elevated in the bilateral LGN and confined to the LGN layers receiving input from the affected eye [7], [8]. On the other hand, Lam et al. [24] have reported that GFAP immunoreactivity was elevated in the LGN layers receiving input from the treated eye after unilateral optic nerve transection or ocular hypertension in monkeys, but found no microglial activation with Mac-1 immunoreactivity in the LGN after ocular hypertension. In this study, we used an antibody which recognized Iba-1 as a microglia marker, and demonstrated microglia activation in the bilateral LGN receiving input from the laser-treated eye as well. Iba-1 is specifically expressed in macrophages/microglia and is upregulated during the activation of these cells. Unlike CR3/43 or Mac-1, Iba-1 is also expressed in ramified microglia (resting form). Therefore, this antibody could be highly sensitive and morphologically distinguish between activated and resting microglia. Accordingly, this discrepancy may be due to the differences of sensitivity among antibodies used in each experiment, but further study is needed. Nevertheless, the findings suggest that the glial activation may become a promising hallmark for early diagnosis and determination of severity of glaucoma.

A non-invasive PET imaging with [^11^C]PK11195 for activated microglial cells has been extensively used in several neurodegenerative disorders, such as multiple sclerosis [25], Alzheimer disease [26], Parkinson disease [27], and Huntington disease [28]. Although PBR is expressed at low levels in resting microglial cells and astrocytes of the normal brain, it is upregulated in activated microglial cells and astrocytes [11], [12], [13]. Previously, we found that [^11^C]PK11195 binding potential value in PET imaging was detected in the bilateral LGN during the advanced glaucoma stage (4 to 12 months) after unilateral ocular hypertension in cynomolgus monkeys [8]. In the present study, we could detect the increase of [^11^C]PK11195 binding potential value in the LGN during the moderate stage of glaucoma after unilateral ocular hypertension. Briefly, [^11^C]PK11195 binding potential values in 4 cynomolgus monkeys were significantly increased in the ipsilateral LGN at 4 weeks after unilateral ocular hypertension compared to before the laser treatment. In the contralateral LGN, [^11^C]PK11195 binding potential values tended to increase, but the differences were not statistically significant. We previously reported that metabolic activity using 2-[^18^F]fluoro-2-deoxy-glucose (^18^FDG) uptake during monocular viewing was significantly reduced in the ipsilateral visuocortical areas of the glaucoma eye [8]. Taken together, this asymmetry might suggest that visual field defects in the nasal area and the corresponding RGC and optic nerve damage in the temporal retina are more severe than are those on the opposite side.

In the present study, we demonstrated for the first time that PBR-positive cells occurred in the LGN layers (including both magnocellular- and parvocellular-layers) that received input from laser-treated eyes at all glaucoma stages. Interestingly, PBR expression was even observed at the mild glaucoma stage with slight increase of disc cupping based on ophthalmoscopy. A portion of the PBR-expressing cells were also with Iba-1-positive microglial cells and GFAP-positive astroglial cells. These data provide further evidence that PBR is persistently expressed in glial cells of the LGN that receive input from the laser-treated eye in mild through advanced stages of glaucoma. In contrast to the asymmetry seen for [^11^C]PK11195 binding potential values between bilateral LGN, Iba-1- , GFAP-, or PBR-positive glial cells showed no similar asymmetry in the bilateral LGN. In the PET imaging, living monkeys were used and, therefore, some differences of biological or haemodynamic activities such as cellular activity and blood flow between the bilateral LGN might be affected to PK11195 binding potentials, but further studies will be needed to explain this discrepancy.

Recently, reduction of functional activity in the primary visual cortex has been reported in open-angle glaucomatous patients with asymmetric visual field damage by using fMRI technique [29], [30], though the result of the correlation between fMRI responses and measurements of optic disc damage is still controversial in these studies. There may be strong plasticity and compensatory mechanism of the visual pathway during early stage of diagnosis progression, which makes difficult to use the activity related fMRI signal for identifying the early indicators of glaucoma. Though MRI scans have generally higher spatial and temporal resolutions than PET, and allows functional assessment of the region brain without any imaging agents, the PET imaging technique has higher sensitivity and specificity to the biological process in the specific cell, PBR in the activated glial cells in this case, which has not been available by MRI technique yet. Therefore, at present PET imaging has more reliable with sensitive and specific values for diagnosing glaucoma especially in the early stage. Furthermore, PET imaging may have more sensitive and specific value for diagnosing glaucoma especially in the early stage.

In conclusion, these findings indicate that non-invasive molecular imaging of the LGN targeted against activated glial markers such as PBR may be useful in diagnosis of glaucoma and in the development of neuroprotective agents. However, in the present study the sample size was small at each stage of glaucoma and, therefore, further additional studies will be needed.
